# Manual of Diseases of the Skin

**Published:** 1852-03

**Authors:** 


*Manual of Diseases of the Skin.* From the French of MM. CAZENAVE & SCHEDEL. With Notes and Additions by THOMAS H. BURGESS, M. D., Surgeon to the Blenheim Street Dispensary for Diseases of the Skin, etc. Second American Edition. Enlarged and corrected, from the last French Edition. With additional Notes, by H. D. BULKLEY, M. D., Physician of the New York Hospital, etc. etc. etc. New York: Published by Samuel S. & William Wood, 261 Pearl-st. 1852.

This is eminently a practical work, and we know of no treatise on skin diseases better suited to the wants of the general practitioner. The classification is on the basis of that of Willan, which will always be retained, as it possesses advantages superior to those of the new arrangement proposed by Wilson. The neglect of the cutanei by the profession would be less general than it is, if the ease and satisfaction with which this province of medicine may be studied, were better known. Many are repelled from it, as from physical exploration, by the impression that the pursuit is complicated and difficult. This impression does great injustice to the subject. It is curious that the two departments of medical practice in which the principles of diagnosis are most capable of being brought to the perfection of demonstration, should be the most neglected.

Dr. Bulkley has added some valuable notes to the present edition.

